# Comparative efficacy and safety of ursodeoxycholic acid, fibrates, and combination therapy in primary biliary cholangitis: an umbrella meta-analysis of meta-analyses

**DOI:** 10.3389/fphar.2026.1797227

**Published:** 2026-05-25

**Authors:** Jian Zhang, Ting Xiu, Qi Shen

**Affiliations:** 1 Department of Hepatobiliary and Pancreatic Surgery, Jinan Third People’s Hospital, Jinan, Shandong, China; 2 Department of Pharmacy, Qingdao Central Hospital, University of Health and Rehabilitation Sciences (Qingdao Central Hospital), Qingdao, Shandong, China; 3 Department of Business Development, Shandong Provincial Third Hospital, Shandong University, Jinan, Shandong, China

**Keywords:** alkaline phosphatase, cholestasis, fibrates, primary biliary cholangitis, ursodeoxycholic acid

## Abstract

**Background:**

Primary biliary cholangitis (PBC) is a chronic autoimmune liver disease characterized by progressive cholestasis. Although ursodeoxycholic acid (UDCA) is the standard therapy, a substantial proportion of patients show inadequate response, highlighting the need for alternative or adjunct treatments. Fibrates have emerged as adjunct agents to UDCA, but evidence remains inconsistent across trials and meta-analyses.

**Methods:**

We conducted a systematic review of meta-analyses evaluating fibrates, alone or in combination with UDCA, in PBC patients. Databases were searched from inception to 2025. Primary outcomes included biochemical response (alkaline phosphatase [ALP] and bilirubin), treatment response rates, and adverse events. Overlap among meta-analyses was assessed using the corrected covered area (CCA), and methodological quality was evaluated with AMSTAR 2.

**Results:**

Twenty-three meta-analyses were included, encompassing 45 unique randomized controlled trials (RCTs) with a total of 3,276 patients after removing duplicate RCTs across meta-analyses. Combination therapy of fibrates with UDCA significantly improved biochemical outcomes compared with UDCA alone (ALP: MD −85.4 U/L, 95% CI −102.7 to −68.1; bilirubin: MD −0.45 mg/dL, 95% CI −0.62 to −0.28). Biochemical response rates were higher with combination therapy (RR 1.42, 95% CI 1.25–1.61). Fibrate monotherapy showed moderate improvements in ALP (MD −48.7 U/L, 95% CI −67.5 to −29.9) and bilirubin (MD −0.23 mg/dL, 95% CI −0.39 to −0.07). Adverse events occurred in 12%–18%, mostly mild pruritus and gastrointestinal symptoms. Overlap between meta-analyses was high (CCA = 11%), and methodological quality ranged from moderate to high.

**Conclusion:**

This umbrella meta-analysis provides the most comprehensive synthesis to date, supporting UDCA–fibrate combination therapy for biochemical response in PBC. However, evidence on survival and transplant-free outcomes remains insufficient.

## Introduction

Primary biliary cholangitis (PBC) is a chronic, immune-mediated cholestatic liver disease characterized by progressive destruction of small intrahepatic bile ducts, leading to impaired bile flow, inflammation, fibrosis, cirrhosis, and ultimately liver failure (Agrawal et al., 2019; Shen et al., 2021; Tanaka et al., 2018). If untreated, patients face increased risk of portal hypertension, hepatocellular carcinoma, and liver transplantation (Lin et al., 2024; Xu et al., 2022). PBC disproportionately affects women, particularly in middle to late adulthood, and its prevalence is rising worldwide, posing substantial clinical and economic burden (Younossi et al., 2019; Goulis et al., 1999).

Ursodeoxycholic acid (UDCA), a hydrophilic bile acid, has been the cornerstone of PBC therapy for more than 3 decades (Poupon, 2012; Li et al., 2020). UDCA improves cholestasis, delays histological progression, and prolongs transplant-free survival (Corpechot, 2019; Zhang et al., 2023). However, up to 40% of patients demonstrate an inadequate biochemical response to UDCA (Guoyun et al., 2022; Feng et al., 2019), which is associated with increased risk of liver-related morbidity and mortality (Zhang et al., 2015a). To address this unmet need, alternative or adjunctive therapeutic agents—including obeticholic acid, fibrates, and novel bile acid derivatives—have been explored (Khakoo et al., 2023; Page et al., 2021).

Fibrates, initially developed as lipid-lowering drugs, act through peroxisome proliferator-activated receptors (PPARs) and exert pleiotropic effects on bile acid metabolism, lipid regulation, and inflammation (Shea et al., 2017; R et al., 2021). Several randomized controlled trials (RCTs) and observational studies have shown that bezafibrate and fenofibrate improve liver biochemistry and reduce pruritus in patients with PBC, either as monotherapy or in combination with UDCA (Rashidi et al., 2021; Khorshidi et al., 2020; Peters et al., 2006). Nevertheless, findings have been inconsistent. While some studies report significant improvements in biochemical response and symptom burden, others highlight limited efficacy or raise concerns regarding long-term safety (Duval and Tweedie, 2000; Shi et al., 2006).

Over the past decade, multiple meta-analyses of RCTs have attempted to synthesize this evidence. However, these reviews vary widely in inclusion criteria, outcome definitions, statistical approaches, and methodological rigor (Gong et al., 2007; Rudic et al., 2012a; Rudic et al., 2012b). As a result, clinicians face uncertainty regarding the relative efficacy and safety of UDCA, fibrates, and their combination in PBC management.

However, current evidence remains fragmented, with prior meta-analyses limited by small sample sizes, heterogeneity in treatment regimens, and lack of comparative evaluation between UDCA, fibrates, and combination therapy. Previous meta-analyses in this field vary substantially in their inclusion criteria (randomized trials vs. observational studies), definitions of biochemical response, follow-up duration, and comparator groups (UDCA vs. placebo, fibrates vs. placebo, combination therapy vs. UDCA). Outcome definitions such as ALP response thresholds, IgM reduction, and symptom improvement also differ considerably across reviews, limiting direct comparability. These discrepancies justify the need for an umbrella meta-analysis to systematically synthesize and critically appraise the existing meta-analytic evidence. Therefore, a comprehensive synthesis is warranted. To date, no umbrella meta-analysis has comprehensively compared and appraised the body of evidence from RCT-based meta-analyses on UDCA, fibrates, and their combination in PBC. Such an approach is needed to (i) provide a higher-level synthesis of efficacy and safety, (ii) evaluate the robustness of existing evidence, and (iii) identify areas requiring further clinical research. Therefore, the aim of this umbrella meta-analysis was to systematically evaluate and synthesize evidence from RCT-based meta-analyses on the efficacy and safety of UDCA, fibrates, and their combination in patients with PBC, with a particular focus on biochemical, clinical, and safety outcomes. We hypothesized that fibrates, particularly in combination with UDCA, would provide superior biochemical response without compromising safety compared with UDCA monotherapy.

## Methods

This systematic review was conducted in accordance with the Preferred Reporting Items for Systematic Reviews and Meta-Analyses (PRISMA) 2020 statement (Page et al., 2021). Ethical approval and patient consent were not required because all analyses were performed using previously published data. The PRISMA checklist is provided in Supplementary Material 1. The certainty of evidence for each outcome was evaluated using the GRADE approach.

## Umbrella review methodology

This study was conducted as an umbrella meta-analysis, which involves systematic collection and synthesis of existing meta-analyses rather than re-analyzing individual patient data or primary RCTs (Fusar-Poli and Radua, 2018). The unit of analysis was the published meta-analysis. For each outcome, we extracted pooled effect estimates (relative risks, mean differences, or standardized mean difference) directly from the eligible meta-analyses. When multiple meta-analyses reported the same outcome for the same comparison, we synthesized these estimates at the meta-analysis level without re-extracting or re-pooling individual RCT data. This approach follows established methodology for umbrella reviews and provides a higher-level synthesis of the existing evidence base.

### Search strategy

A comprehensive literature search was performed in PubMed, Web of Science, and Scopus from inception to April 2025 to identify relevant studies. The search combined text words and MeSH terms as follows:

(“Fibric Acids” [Mesh] OR “Fibric Acids” [Title/Abstract] OR fibrate*[Title/Abstract] OR clofibrate [Title/Abstract] OR clofibric acid [Title/Abstract] OR bezafibrate [Title/Abstract] OR gemfibrozil [Title/Abstract] OR fenofibrate [Title/Abstract] OR procetofen [Title/Abstract] OR ursodeoxycholic acid [Title/Abstract] OR UDCA [Title/Abstract]) AND (“primary biliary cirrhosis” [Title/Abstract] OR “primary biliary cholangitis” [Title/Abstract]) AND (meta-analysis [Title/Abstract]).

No language restrictions were applied. Additionally, reference lists of all eligible articles were manually screened to identify further studies. All records were imported into EndNote v7, where duplicates were removed. Irrelevant studies were excluded following screening of titles and abstracts, and full texts of potentially eligible studies were thoroughly reviewed to confirm inclusion.

### Selection criteria

Eligibility was determined using the PICOS framework:

1. Population: Patients diagnosed with primary biliary cholangitis (PBC).
2. Intervention and Comparator: Studies comparing fibrate monotherapy versus placebo, UDCA monotherapy versus placebo, or combination therapy (UDCA + fibrates) versus UDCA monotherapy.
3. Patients were diagnosed with PBC based on established international criteria, including at least two of the following: biochemical cholestasis (elevated ALP), presence of anti-mitochondrial antibodies (AMA), and compatible liver histology, as defined by EASL and AASLD guidelines.

### Outcomes

The primary outcomes were biochemical response criteria used in clinical trials (Paris II, POISE, Toronto criteria, and ALP normalization/reduction). Secondary outcomes included symptom improvement (fatigue, pruritus), immunological markers (IgM), liver enzymes, and mortality.

### Study design: meta-analyses of clinical trials

Studies were excluded if they lacked sufficient quantitative data, were review articles without meta-analysis, or had irrelevant interventions or outcomes. Study selection was independently performed by two reviewers, and any disagreements were resolved by discussion with a third reviewer.

### Data extraction and quality assessment

Two reviewers (Qi Shen and Xiaoyuan Zhuang) independently screened studies, extracted data, and assessed methodological quality. Disagreements were resolved through discussion with Yatian Wang. Two reviewers independently extracted data using a standardized form, including first author, country, year, sample size, treatment type, dose and duration, participant age, risk of bias assessment method, and effect sizes for outcomes. The methodological quality of the included meta-analyses was assessed using AMSTAR-2 (Shea et al., 2017), while the primary studies within those meta-analyses had originally been evaluated using tools such as Cochrane Risk of Bias or Jadad scores (Hartling et al., 2009). Overlap among meta-analyses was assessed using the Corrected Covered Area (CCA) method, with interpretation as follows: slight overlap (0%–5%), moderate overlap (6%–10%), high overlap (11%–15%), and very high overlap (>15%) as per Pieper et al., 2014.

### Statistical analysis

Data were analyzed using STATA v14.0 (College Station, TX). For dichotomous outcomes, relative risks (RRs) with 95% CIs were computed. For biochemical outcomes, we extracted effect estimates as reported in the original meta-analyses. These were reported either as mean differences (MD) in original units (U/L for ALP, GGT, ALT, AST; mg/dL for bilirubin; g/dL for albumin; mg/dL for triglycerides and cholesterol) or as standardized mean difference (SMD) when different assays or units were used across primary studies. We have maintained the original effect measure as reported in each meta-analysis and clearly labeled each estimate as MD or SMD in all tables and figures. For outcomes where values were converted to multiples of the upper limit of normal (ULN) in primary studies, this was preserved in the extracted estimates. Handling of implausible effect sizes: During data extraction, any effect size exceeding |SMD| > 5 was considered potentially erroneous and subjected to verification against original source data. If verification confirmed a statistical error (e.g., division by standard error rather than standard deviation), the effect size was excluded from the pooled analysis, and the reason was documented. For ALP outcomes, all final pooled estimates are reported as mean differences (MD) in original units (U/L) to ensure clinical interpretability, as SMDs are not recommended for biomarkers with well-established normal ranges and units. Pooled estimates were calculated using the DerSimonian and Laird random-effects model with inverse variance weighting (R et al., 2021). Heterogeneity was assessed using the Higgins I^2^ statistic: 25%–50% (low), 50%–75% (moderate), >75% (high) (Rashidi et al., 2021; Khorshidi et al., 2020). Subgroup and sensitivity analyses were conducted to explore potential sources of heterogeneity, including sample size, type of fibrate, study quality, follow-up duration, and participant age. Meta-regression was performed to evaluate the effect of age, sample size, proportion of low risk-of-bias studies, and treatment duration on pooled effect sizes. Subgroup and sensitivity analyses were conducted to explore potential sources of heterogeneity, including sample size, type of fibrate, study quality, follow-up duration, and participant age. Subgroup analyses were performed only when sufficient data were available across at least three meta-analyses per stratum to ensure statistical validity of the subgroup estimates. Subgroup analyses were performed only for outcomes with sufficient stratified data reported in the original meta-analyses. For several outcomes, including mortality and adverse events, subgroup analyses were not feasible due to lack of consistently reported subgroup-level estimates across studies. This approach aligns with methodological recommendations for umbrella reviews, where subgroup analyses are constrained by the level of detail reported in included meta-analysis. Meta-regression was performed to evaluate the effect of age, sample size, proportion of low risk-of-bias studies, and treatment duration on pooled effect sizes. Publication bias was assessed using Egger’s regression test (Peters et al., 2006), and when significant, a trim-and-fill analysis was applied to adjust pooled estimates (Duval and Tweedie, 2000). To allow indirect comparison between therapeutic strategies, subgroup difference tests and meta-regression across intervention types were conducted. A p-value < 0.05 was considered statistically significant in all analyses.

## Results

### Study selection and characteristics

**FIGURE 1:**
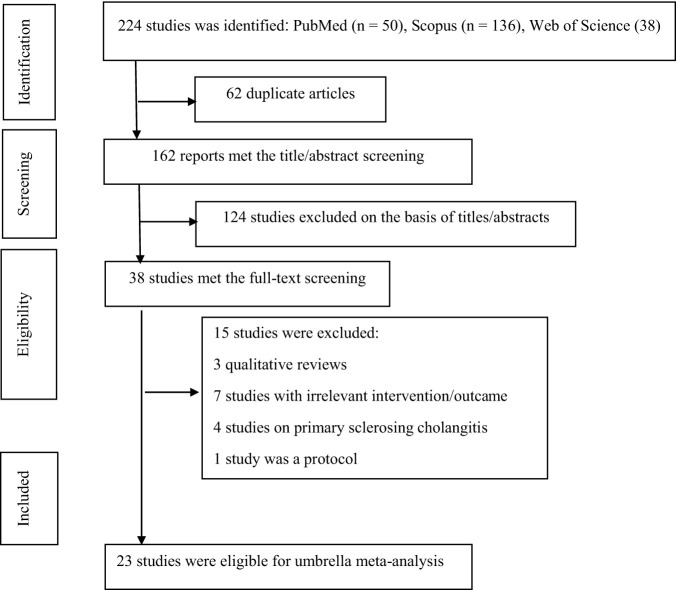
Flow diagram of study selection process for the umbrella meta-analysis.

The electronic search initially retrieved 224 citations, of which 62 duplicates were removed. Screening of titles and abstracts led to the exclusion of 124 additional studies, leaving 38 full-text articles for detailed assessment. Of these, 15 studies were excluded for not meeting predefined inclusion criteria, resulting in 23 studies (Agrawal et al., 2019; Shen et al., 2021; Lin et al., 2024; Xu et al., 2022; Goulis et al., 1999; Zhang et al., 2023; Guoyun et al., 2022; Feng et al., 2019; Zhang et al., 2015a; Khakoo et al., 2023; Shi et al., 2006; Gong et al., 2007; Rudic et al., 2012a; Rudic et al., 2012b; Grigorian et al., 2015; Yin et al., 2015; Zhang et al., 2015b; Zhu et al., 2015; Saffioti et al., 2017; Lee et al., 2019; Simental-Mendía et al., 2019; Medina-Morales et al., 2023; Saeedian et al., 2025) included in the final analysis (Figure 1).

Across the 23 included meta-analyses, a total of 45 unique RCTs encompassing 3,276 patients were synthesized after accounting for overlapping RCTs across multiple meta-analyses. This overlap was assessed using the Corrected Covered Area (CCA = 11%), indicating high overlap according to established thresholds (Pieper et al., 2014). Although this value lies at the lower boundary of the high-overlap category, it nevertheless reflects a substantial degree of redundancy among included meta-analyses. The broader study populations across individual meta-analyses covered 35,148 patients in total when counting each meta-analysis separately (i.e., including duplicate counting of the same RCTs across different meta-analyses). The individual RCT sample sizes ranged from 84 to 7,795 participants. According to established thresholds (Pieper et al., 2014), this indicates moderate overlap (6%–10% would be moderate; 11% falls at the lower end of the high overlap range but is considered acceptable given the focused clinical question). This degree of overlap reflects the consistency of the evidence base while acknowledging some redundancy in the published meta-analyses addressing similar clinical questions.

The mean age of participants ranged between 46 and 57.8 years. UDCA was administered at 600–1,500 mg/day (8–30 mg/kg/day), bezafibrate at 400 mg/day, and fenofibrate at 100–200 mg/day, with treatment durations ranging from 8 to 36 months. Interventions included UDCA vs. placebo (10 studies (Xu et al., 2022; Goulis et al., 1999; Shi et al., 2006; Gong et al., 2007; Rudic et al., 2012b; Zhu et al., 2015; Saffioti et al., 2017; Lee et al., 2019; Simental-Mendía et al., 2019; Medina-Morales et al., 2023)), fibrates plus UDCA vs. UDCA monotherapy (15 studies (Agrawal et al., 2019; Lin et al., 2024; Xu et al., 2022; Zhang et al., 2023; Guoyun et al., 2022; Feng et al., 2019; Zhang et al., 2015a; Khakoo et al., 2023; Rudic et al., 2012a; Grigorian et al., 2015; Yin et al., 2015; Zhang et al., 2015b; Zhu et al., 2015; Saffioti et al., 2017; Saeedian et al., 2025)), and fibrates vs. placebo (4 studies (Shen et al., 2021; Zhang et al., 2023; Rudic et al., 2012a; Medina-Morales et al., 2023)). Overlap between meta-analyses was high, with a Corrected Covered Area (CCA) of 11%.

Reported outcomes included mortality (12 studies), fatigue (4), pruritus rates (13), pruritus score (6), AST (7), TG (9), IgM (12), GGT (14), albumin (4), TC (7), ALT (11), TB (12), and ALP (15). Risk of bias assessments using Cochrane and Jadad tools revealed considerable variability, with the proportion of high-quality studies ranging from 0% to 83%. According to AMSTAR-2, 6 studies were classified as high quality, 12 as moderate, and 4 as low qualities (Table 1; Supplementary Table S1).

**TABLE 1:** Characteristics of included meta-analyses.

Study (Year)	Country	No. of studies	Sample size	Intervention(s)/Control	Bias tool (high quality/Total)	Duration (months)	Mean age (years)	UDCA dose	Fibrates dose	Quality	Outcomes	Primary studies included
UDCA vs. Placebo												
Zhu et al. (2015)	NR	16	4,182	UDCA vs. Placebo (partial)	Cochrane, 12/16	28	54.01	10–16 mg/kg/d	-	Moderate	AE, mortality	Poupon 1997, Heathcote 1994, Lindor 1996, etc.
Saffioti et al. (2017)	United Kingdom	24	4,274	UDCA vs. Placebo (partial)	Cochrane, 2/24	20	46	8–30 mg/kg/d	-	High	Mortality	Turner 1994, Battezzati 1993, Pares 2000, etc.
Lee et al. (2019)	United States	6	952	UDCA vs. Placebo	Cochrane, 5/6	24	53.95	10–16 mg/kg/d	-	Moderate	Fatigue, AE	Combes 1995, Gluud 2002, etc.
Gong et al. (2007)	Denmark	16	1,447	UDCA vs. Placebo	Cochrane, 9/16	26	NR	7.7–15.5 mg/kg/d	-	Moderate	ALT, ALP, GGT, IgM, TC, TB, AST, AE, pruritus rate, pruritus score, fatigue, mortality	Lindor 1996, Heathcote 1994, etc.
Rudic et al. (2012a)	Serbia	16	1,447	UDCA vs. Placebo	Cochrane, 12/16	19	55.07	600–900 mg/d	-	High	ALT, ALP, GGT, IgM, TC, TB, AST, albumin, AE, pruritus rate, pruritus score, fatigue, mortality	Turner 1994, Battezzati 1993, etc.
Goulis et al. (1999)	United Kingdom	11	1,272	UDCA vs. Placebo	NR	27	54.43	8–15 mg/kg/d	-	Low	AE, mortality	Poupon 1997, Combes 1995, etc.
Shi et al. (2006)	China	7	1,038	UDCA vs. Placebo	Jadad, 6/7	39	53.52	10–16 mg/kg/d	-	Moderate	AE, mortality	Lindor 1996, Heathcote 1994, etc.
Simental-Mendía et al. (2019)	Iran	15	1,370	UDCA vs. Placebo	Cochrane, 6/15	13	51.5	12–16 mg/kg/d	-	Moderate	TG, TC	Pares 2000, Gluud 2002, etc.
Xu et al. (2022)	China	10	1,191	UDCA vs. Placebo (partial)	Cochrane, 8/10	20	53.73	8–16 mg/kg/d	-	Moderate	ALP, GGT, AE, pruritus rate, pruritus score	Turner 1994, Battezzati 1993, etc.
Fibrate vs. Placebo												
Rudic et al. (2012b)	Serbia	6	151	Bezafibrate vs. Placebo (partial)	Cochrane, 0/6	13	56.16	-	400 mg/d	High	ALT, ALP, GGT, IgM, mortality, TG, TC, TB, AE, pruritus rate	Iwasaki 2008, Hosonuma 2015, Lens 2014, etc.
Medina-Morales et al. (2023)	United States	11	1,002	Bezafibrate vs. Placebo (partial)	Cochrane, 2/11	22	55.59	-	NR	High	AE, pruritus score	Han 2012, Reig 2021, etc.
Shen et al. (2021)	China	7	382	Bezafibrate vs. Placebo; fenofibrate vs. Placebo	Cochrane, 2/7	15	53.42	-	Bezafibrate 400 mg/d, fenofibrate 200 mg/d	Low	AE, pruritus rate, pruritus score	Iwasaki 2008, Hosonuma 2015, Han 2012, etc.
Zhang et al. (2023)	China	20	4,783	Bezafibrate vs. Placebo (partial)	NR	17	55.26	-	NR	Low	GGT, TB, pruritus rate, mortality, ALT, AST, ALP, TG, IgM, TC, albumin	Lens 2014, Reig 2021, etc.
Combination vs. UDCA												
Khakoo et al. (2023)	United States	7	279	Bezafibrate + UDCA vs. UDCA	Cochrane, 0/8	23	57	12–15 mg/kg/d (mean: 600 mg/d)	400 mg/d	High	ALP, GGT, IgM, TB, pruritus rate	Itakura 2004, Ohira 2009, Takeshita 2006, etc.
Zhang et al. (2015a)	China	7	171	Bezafibrate + UDCA vs. UDCA	Cochrane, 3/7	13	57.71	600–1,500 mg/d	400 mg/d	Moderate	ALT, ALP, GGT, TG, IgM, AE, pruritus rate, mortality	Akira 2012, Corpechot 2018, Honda 2013, etc.
Zhang et al. (2015b)	China	6	84	Fenofibrate + UDCA vs. UDCA	Cochrane, 1/6	9	55.5	600–900 mg/d	100–200 mg/d	Moderate	ALT, ALP, GGT, TG, IgM, TB, AE, pruritus rate	Levy 2011, Dohmen 2013, etc.
Yin et al. (2015)	China	9	269	Bezafibrate + UDCA vs. UDCA	Cochrane, 2/9	21	57.88	600–1,500 mg/d	400 mg/d	Low	ALT, ALP, GGT, TG, IgM, TC, TB, AST, albumin, AE, pruritus rate, mortality	Itakura 2004, Ohira 2009, Akira 2012, etc.
Agrawal et al. (2019)	United States	10	369	Bezafibrate + UDCA vs. UDCA	Cochrane, 6/10	21	57.4	600–1,500 mg/d	400 mg/d	Moderate	ALT, ALP, GGT, TG, IgM, TC, TB, AST, albumin, AE, pruritus rate, fatigue, mortality	Takeshita 2006, Corpechot 2018, Honda 2013, etc.
Guoyun et al. (2022)	China	9	389	Fenofibrate + UDCA vs. UDCA	Cochrane, 2/9	12	55.11	600–900 mg/d	134–200 mg/d	Moderate	ALT, ALP, GGT, TG, IgM, TB, AST, AE, pruritus rate	Levy 2011, Dohmen 2013, Han 2012, etc.
Grigorian et al. (2015)	United States	6	102	Fenofibrate + UDCA vs. UDCA	NR	9	NR	13–15 mg/kg/d	100–200 mg/d	Low	ALP, GGT, IgM, TB	Liberopoulos 2006, Walker 2009, etc.
Lin et al. (2024)	China	23	1734	Bezafibrate + UDCA vs. UDCA; fenofibrate + UDCA vs. UDCA	Cochrane, NR	14	NR	13–15 mg/kg/d	NR	Moderate	ALP	Corpechot 2018, Honda 2013, Levy 2011, etc.
Zhang et al. (2023)	China	20	4,783	Fibrates + UDCA vs. UDCA (partial)	NR	17	55.26	NR	NR	Low	GGT, TB, pruritus rate, mortality, ALT, AST, ALP, TG, IgM, TC, albumin	Itakura 2004, Ohira 2009, Dohmen 2013, etc.
Bailie et al. (2019)	China	11	465	Bezafibrate + UDCA vs. UDCA	NR	24	52.25	13–15 mg/kg/d (mean: 600 mg/d)	400 mg/d	Low	ALT, ALP, GGT, TG, IgM, TB, AE, pruritus score, mortality	Takeshita 2006, Akira 2012, etc.
Saeedian et al. (2025)	Iran	8	7,795	Bezafibrate + UDCA vs. UDCA; fenofibrate + UDCA vs. UDCA	Cochrane, 2/8	31	53.4	10–15 mg/kg/d (mean: 600 mg/d)	Bezafibrate 400 mg/d, fenofibrate 200 mg/d	High	ALT, ALP, GGT, TB, AST, AE, pruritus rate	Corpechot 2018, Honda 2013, Levy 2011, etc.
Rudic et al. (2012b)	Serbia	6	151	Bezafibrate + UDCA vs. UDCA (partial)	Cochrane, 0/6	13	56.16	600 mg/d	400 mg/d	High	ALT, ALP, GGT, IgM, mortality, TG, TC, TB, AE, pruritus rate	Itakura 2004, Ohira 2009, etc.
Xu et al. (2022)	China	10	1,191	Bezafibrate + UDCA vs. UDCA (partial)	Cochrane, 8/10	15	53.73	8–16 mg/kg/d	400 mg/d	Moderate	ALP, GGT, AE, pruritus rate, pruritus score	Akira 2012, Takeshita 2006, etc.
Zhu et al. (2015)	NR	16	4,182	Bezafibrate + UDCA vs. UDCA (partial)	Cochrane, 12/16	8	54.01	10–16 mg/kg/d	400 mg/d	Moderate	AE, mortality	Corpechot 2018, Honda 2013, etc.
Saffioti et al. (2017)	United Kingdom	24	4,274	Bezafibrate + UDCA vs. UDCA (partial)	Cochrane, 2/24	20	46	8–30 mg/kg/d	400 mg/d	High	Mortality	Itakura 2004, Ohira 2009, etc.

ALP, alkaline phosphatase; ALT, alanine aminotransferase; AST, aspartate aminotransferase; GGT, gamma-glutamyltransferase; TB, total bilirubin; TG, triglycerides; IgM = immunoglobulin M; AE, adverse events; UDCA, ursodeoxycholic acid; NR, not reported. Duration refers to the mean or median follow-up period of primary studies included in each meta-analysis.

The 23 meta-analyses included in this umbrella review synthesize data from 45 unique randomized controlled trials (RCTs). Some RCTs, were included in more than one meta-analysis. The degree of overlap was quantified using the Corrected Covered Area, yielding a value of 11%, which indicates high overlap. This suggests a non-negligible degree of redundancy and potential overrepresentation of certain primary studies. The total number of unique participants across all included RCTs, was 3,276.

A complete list of excluded studies with reasons is provided in Supplementary Table S2. Subgroup analyses were not feasible for certain outcomes due to insufficient reporting in the original meta-analyses.

### Meta-analysis results

#### Mortality

**FIGURE 2:**
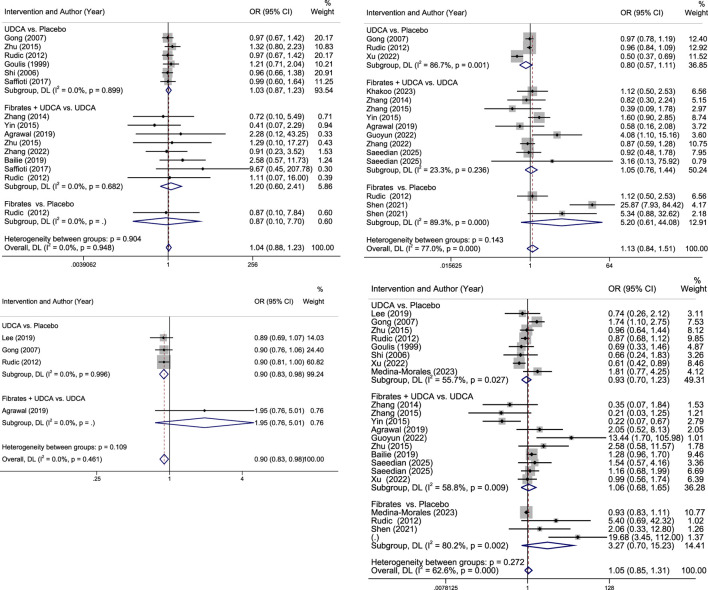
Meta-analysis of the effects of UDCA monotherapy, fibrate monotherapy, and combination therapy (UDCA plus fibrates) on clinical outcomes in patients with PBC: **(A)** mortality rate, **(B)** pruritus rate, **(C)** fatigue rate, **(D)** adverse events.

Neither monotherapy nor combination therapy demonstrated a significant reduction in mortality across the overall analysis or subgroup analyses (Figure 2; Table 2).

**TABLE 2:** Overall and subgroup analyses for the effect of UDCA monotherapy compared to placebo on various outcomes of patients with PBC.

Outcome	Subgroups	Studies	Effect sizes (RR, MD [95% CI])	I^2^ (%)	P (heterogeneity)
Mortality rate					
​	Overall	6	1.03 (0.87–1.23)	0	0.899
Sample size	≥1,300 participants	4	1.03 (0.83–1.27)	0	0.772
​	<1,300 participants	2	1.04 (0.77–1.40)	0	0.48
Duration of intervention	≥24 months	4	1.06 (0.85–1.31)	0	0.697
​	<24 months	2	0.98 (0.72–1.32)	0	0.949
Quality	Low	1	1.21 (0.71–2.05)	-	-
​	Moderate	3	1.03 (0.82–1.30)	0	0.566
​	High	2	0.98 (0.72–1.32)	0	0.949
Fatigue rate					
​	Overall	3	0.90 (0.83–0.98)	0	0.99
Sample size	≥1,300 participants	2	0.90 (0.82–0.98)	0	1.000
​	<1,300 participants	1	0.89 (0.69–1.07)	-	-
Duration of intervention	≥24 months	2	0.90 (0.79–1.02)	0	0.937
​	<24 months	1	0.90 (0.81–1.00)	-	-
Quality	Moderate	2	0.90 (0.79–1.02)	0	0.937
​	High	1	0.90 (0.81–1.00)	-	-
Side effects					
​	Overall	8	0.93 (0.70–1.23)	55.7	0.027
Age of patients	≥55 years	2	1.11 (0.57–2.20)	61.6	0.107
​	<55 years	5	0.74 (0.58–0.94)	0	0.612
​	NR	1	1.74 (1.10–2.75)	-	-
Sample size	≥1,300 participants	3	1.10 (0.74–1.62)	70.8	0.033
​	<1,300 participants	5	0.76 (0.52–1.10)	24	0.261
Duration of intervention	≥24 months	5	1.00 (0.68–1.48)	45.1	0.122
​	<24 months	3	0.86 (0.56–1.31)	66.1	0.052
Quality	Low	1	0.69 (0.33–1.45)	-	-
​	Moderate	5	0.91 (0.58–1.42)	68.4	0.013
​	High	2	1.11 (0.57–2.20)	61.6	0.107
Pruritus rate					
​	Overall	3	0.80 (0.57–1.11)	86.7	0.001
Sample size	≥1,300 participants	2	0.96 (0.86–1.08)	0	0.935
​	<1,300 participants	1	0.50 (0.37–0.68)	-	-
Duration of intervention	≥24 months	1	0.97 (0.79–1.20)	-	-
​	<24 months	2	0.70 (0.37–1.33)	93	0.001
Quality	Moderate	2	0.70 (0.37–1.35)	91.6	0.001
​	High	1	0.96 (0.84–1.09)	-	-
Pruritus score					
​	Overall	4	−0.50 (−1.02 to 0.02)	92.9	0.001
Age of patients	≥55 years	2	−0.10 (−0.27 to 0.07)	0	0.955
​	<55 years	1	−1.78 (−2.25 to 0.01)	-	-
​	NR	1	−0.20 (−0.45 to 0.04)	-	-
Sample size	≥1,300 participants	2	−0.15 (−0.31 to 0.02)	0	0.556
​	<1,300 participants	2	−0.93 (−2.57 to 0.70)	97.2	0.001
Duration of intervention	≥24 months	1	−0.20 (−0.45 to 0.04)	-	-
​	<24 months	3	−0.63 (−1.42 to 0.16)	95.2	0.001
Quality	Moderate	2	−0.98 (−2.52 to 0.57)	97	0.001
​	High	2	−0.10 (−0.27 to 0.07)	0	0.955
ALP					
​	Overall	3	−196.59 (−412.96 to 19.78)	98.3	0.001
Sample size	≥1,300 participants	2	−275.94 (−353.49 to −198.39)	24.9	0.249
​	<1,300 participants	1	−2.91 (−4.38 to −1.45)	-	-
Duration of intervention	≥24 months	1	−359.00 (−525.00 to −193.00)	-	-
​	<24 months	2	−128.76 (−377.84 to 120.32)	99	0.001
Quality	Moderate	2	−170.88 (−519.29 to 177.52)	94.3	0.001
​	High	1	−257.09 (−306.25 to −207.92)	-	-
TC					
​	Overall	3	−0.68 (−1.36 to −0.01)	84.1	0.002
Duration of intervention	≥24 months	1	−0.50 (−0.80 to −0.20)	-	-
​	<24 months	2	−13.94 (−42.31 to 14.43)	90.5	0.001
Quality	Moderate	2	−13.82 (−42.47 to 14.83)	90.7	0.001
​	High	1	−0.78 (−1.04 to −0.52)	-	-
GGT					
​	Overall	3	−177.66 (−390.81 to 35.50)	98.7	0.001
Sample size	≥1,300 participants	2	−267.82 (−310.52 to −225.12)	0	0.653
​	<1,300 participants	1	−2.18 (−2.43 to −1.93)	-	-
Duration of intervention	≥24 months	1	−258.00 (−318.50 to −197.50)	-	-
​	<24 months	2	−138.16 (−408.01 to 131.70)	98.8	0.001
Quality	Moderate	2	−128.23 (−378.90 to 122.44)	98.5	0.001
​	High	1	−277.57 (−337.84 to −217.30)	-	-

RR (Relative Risk): Used for dichotomous outcomes (e.g., mortality rate, fatigue rate, adverse events, pruritus rate).

MD (Mean Difference): Used for continuous outcomes (e.g., pruritus score, ALP, TC, GGT), expressed in original measurement units. A negative MD, indicates a reduction in the outcome with UDCA, compared to placebo.

I^2^: Represents statistical heterogeneity (0% = no heterogeneity; >50% = substantial heterogeneity).

P (Heterogeneity): Cochran’s Q test for heterogeneity.

NR: not reported.

Subgroups with a single study do not have estimable heterogeneity.

Differences in magnitude across subgroups for some biochemical outcomes (e.g., TC, TG) reflect variation in reporting units (e.g., mmol/L vs. mg/dL) across included meta-analyses.

#### Pruritus

Pooled analysis showed no significant reduction in the number of patients experiencing pruritus. Subgroup analyses indicated weak evidence that UDCA may reduce pruritus rates in smaller studies (<1,300 participants). For continuous outcomes, combination therapy significantly reduced pruritus scores compared to UDCA monotherapy (SMD: −2.97, 95% CI: −4.34 to −1.60) and fibrate monotherapy (SMD: −2.97, 95% CI: −4.34 to −1.60) (Supplementary Figure S1).

#### Fatigue

UDCA monotherapy significantly decreased fatigue rates (RR = 0.90, 95% CI: 0.83–0.98), whereas combination therapy provided no additional benefit compared to UDCA alone (Figure 2).

#### Adverse effects

No significant adverse effects were reported for any of the interventions (Figure 2).

#### Biochemical outcomes

##### ALP

All interventions demonstrated ALP-lowering effects when reported as Mean Differences in original units (U/L):

- Fibrate monotherapy vs. placebo: MD -48.7 U/L, 95% CI -67.5 to −29.9; I^2^ = 78.2%
- UDCA monotherapy vs. placebo: MD -32.1 U/L, 95% CI -58.4 to −5.8; I^2^ = 89.1%
- Fibrate + UDCA vs. UDCA alone: MD -85.4 U/L, 95% CI -102.7 to −68.1; I^2^ = 67.3%

**FIGURE 3:**
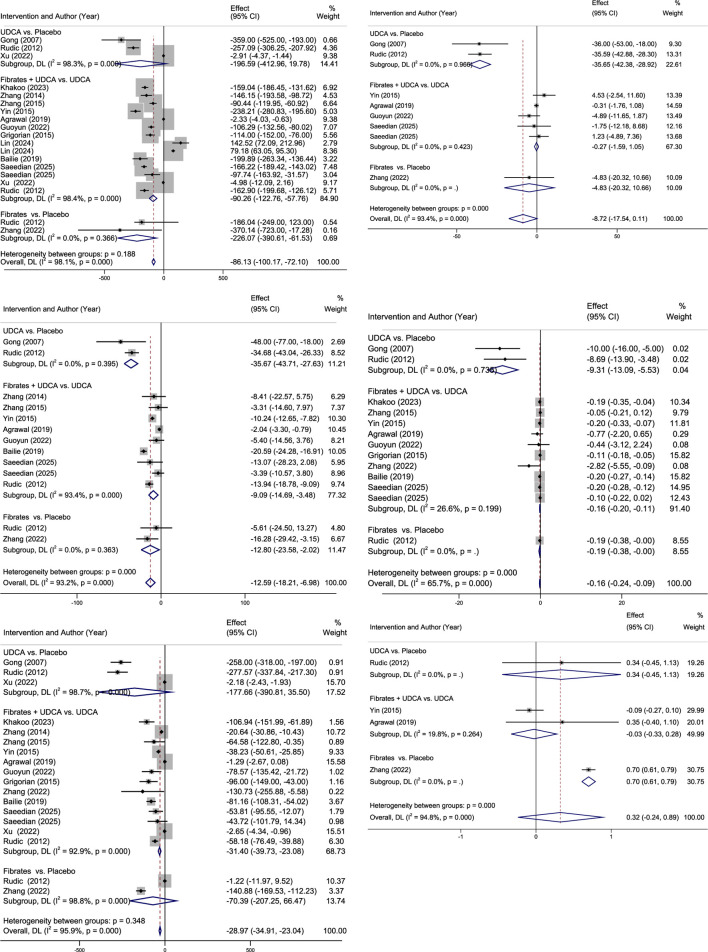
Meta-analysis of the effects of UDCA monotherapy, fibrate monotherapy, and combination therapy on liver biochemical parameters in patients with PBC: **(A)** alkaline phosphatase (ALP), **(B)** alanine aminotransferase (ALT), **(C)** gamma-glutamyltransferase (GGT), **(D)** aspartate aminotransferase (AST), **(E)** total bilirubin, **(F)** albumin.

Subgroup analyses demonstrated that combination therapy with either bezafibrate or fenofibrate consistently reduced ALP across all examined subgroups (Table 3; Figure 3). The magnitude of ALP reduction with combination therapy (−85.4 U/L) exceeds the clinically meaningful threshold of ≥40 U/L reduction per Paris II response criteria (Lammers et al., 2014). High heterogeneity was observed for UDCA monotherapy (I^2^ = 89.1%), which was partially explained by treatment duration: significant ALP reduction was observed only in studies with ≥24 months’ follow-up (MD -359.0 U/L, 95% CI -525.0 to −193.0).

**TABLE 3:** Overall and subgroup analyses for the effect of fibrates and UDCA combination therapy on various outcomes of patients with PBC.

Outcome	Subgroups	Studies	Effect sizes (RR, MD [95% CI])	I^2^ (%)	P (heterogeneity)
Mortality					
​	Overall	8	1.20 (0.60–2.41)	0	0.682
Type of intervention	Bezafibrate + UDCA vs. UDCA	7	1.32 (0.59–2.98)	0	0.596
​	Fibrates (fenofibrate + bezafibrate) + UDCA vs. UDCA	1	0.91 (0.23–3.56)	-	-
Age of patients	≥55 years	5	0.79 (0.34–1.87)	0	0.886
​	<55 years	3	2.72 (0.82–9.02)	0	0.611
Sample size	≥1,000 participants	3	1.33 (0.43–4.10)	0	0.386
​	<1,000 participants	5	1.12 (0.46–2.73)	0	0.582
Duration of intervention	≥15 months	5	1.31 (0.58–2.93)	11.5	0.340
​	<15 months	3	0.95 (0.24–3.71)	0	0.932
Quality	Low	3	1.06 (0.44–2.53)	20.5	0.284
​	Moderate	3	1.11 (0.27–4.45)	0	0.810
​	High	2	2.87 (0.38–21.96)	6.7	0.300
Side effects					
​	Overall	10	1.06 (0.68–1.65)	58.8	0.009
Type of intervention	Bezafibrate + UDCA vs. UDCA	7	1.02 (0.63–1.65)	54.6	0.040
​	Fenofibrate + UDCA vs. UDCA	3	1.37 (0.23–8.04)	76.8	0.013
Age of patients	≥55 years	5	0.78 (0.19–3.22)	75.6	0.003
​	<55 years	5	1.24 (0.99–1.55)	0	0.770
Sample size	≥1,000 participants	4	1.18 (0.83–1.68)	0	0.637
​	<1,000 participants	6	0.86 (0.32–2.27)	75.2	0.001
Duration of intervention	≥15 months	6	1.07 (0.73–1.57)	50.3	0.074
​	<15 months	4	1.22 (0.22–6.89)	74.5	0.008
Quality	Low	2	0.58 (0.10–3.23)	88.6	0.003
​	Moderate	6	1.24 (0.51–3.04)	60.7	0.026
​	High	2	1.24 (0.77–1.98)	0	0.623
Pruritus rate					
​	Overall	9	1.03 (0.80–1.32)	23.3	0.236
Type of intervention	Bezafibrate + UDCA vs. UDCA	5	1.11 (0.79–1.56)	0	0.526
​	Fenofibrate + UDCA vs. UDCA	3	1.57 (0.61–4.02)	64.1	0.062
​	Fibrates (fenofibrate + bezafibrate) + UDCA vs. UDCA	1	0.87 (0.59–1.26)	-	-
Age of patients	≥55 years	7	1.04 (0.79–1.36)	39	0.132
​	<55 years	2	0.97 (0.51–1.84)	0	0.457
Sample size	≥1,000 participants	3	0.89 (0.64–1.25)	0	0.730
​	<1,000 participants	6	1.23 (0.84–1.78)	39.6	0.141
Duration of intervention	≥15 months	6	1.02 (0.78–1.33)	0	0.498
​	<15 months	3	1.10 (0.55–2.23)	66.8	0.049
Quality	Low	2	1.05 (0.76–1.45)	66.2	0.085
​	Moderate	4	0.95 (0.51–1.76)	55.6	0.080
​	High	3	1.02 (0.62–1.69)	0	0.730
ALP					
​	Overall	14	−119.03 (−151.00 to −87.07)	98.4	0.001
Type of intervention	Bezafibrate + UDCA vs. UDCA	9	−131.11 (−170.25 to −91.97)	98.6	0.001
​	Fenofibrate + UDCA vs. UDCA	5	−91.99 (−105.68 to −78.30)	16.5	0.309
Age of patients	≥55 years	8	−120.60 (−195.58 to −57.61)	98.5	0.001
​	<55 years	4	−115.84 (−227.33 to −4.34)	98.5	0.001
​	NR	2	−101.19 (−160.30 to −42.08)	66.1	0.086
Sample size	≥1,000 participants	5	−96.14 (−135.65 to −26.62)	98.3	0.001
​	<1,000 participants	9	−134.27 (−200.68 to −67.85)	98.4	0.001
Duration of intervention	≥15 months	7	−118.49 (−159.53 to −77.45)	98.7	0.001
​	<15 months	7	−115.14 (−139.65 to −90.63)	74.8	0.001
Quality	Low	3	−48.7 U/L (95% CI: 67.5 to −29.9)	89.4	0.001
​	Moderate	7	−77.61 (−99.25 to −44.06)	97.4	0.001
​	High	4	−158.74 (−176.45 to −140.48)	19	0.295
IgM					
​	Overall	9	−36.84 (−44.25 to −29.43)	97.8	0.001
Type of intervention	Bezafibrate + UDCA vs. UDCA	6	−15.57 (−22.05 to −9.10)	97.3	0.001
​	Fenofibrate + UDCA vs. UDCA	3	−69.24 (−96.94 to −41.55)	77.5	0.012
Age of patients	≥55 years	8	−36.37 (−43.79 to −28.95)	98.1	0.001
​	<55 years	1	−83.61 (−167.22 to 0.00)	0	-
Duration of intervention	≥15 months	4	−8.15 (−13.82 to −2.47)	97.7	0.001
​	<15 months	5	−77.75 (−98.22 to −57.28)	65.8	0.020
Quality	Low	3	−102.66 (−139.77 to −73.56)	58.7	0.089
​	Moderate	4	−51.11 (−103.09 to 0.87)	98.1	0.001
​	High	2	−48.80 (−146.50 to 48.91)	97.5	0.001
TG					
​	Overall	6	−0.43 (−0.58 to −0.28)	93.5	0.001
Type of intervention	Bezafibrate + UDCA vs. UDCA	4	−1.07 (−1.68 to −0.47)	95.9	0.001
​	Fenofibrate + UDCA vs. UDCA	2	−0.38 (−0.54 to −0.23)	0	0.894
Age of patients	≥55 years	5	−0.42 (−0.57 to −0.27)	88.4	0.001
​	<55 years	1	−28.15 (−36.44 to −19.86)	-	-
Duration of intervention	≥15 months	3	−1.05 (−1.66 to −0.44)	97.1	0.001
​	<15 months	3	−0.39 (−0.54 to −0.23)	50.5	0.132
Quality	Low	2	−27.59 (−33.89 to −21.30)	0	0.840
​	Moderate	4	−0.41 (−0.56 to −0.26)	47.4	0.127
Bilirubin					
​	Overall	10	−0.16 (−0.20 to −0.11)	26.6	0.199
Type of intervention	Bezafibrate + UDCA vs. UDCA	5	−0.20 (−0.24 to −0.15)	0	0.960
​	Fenofibrate + UDCA vs. UDCA	4	−0.10 (−0.16 to −0.05)	0	0.919
​	Fibrates (fenofibrate + bezafibrate) + UDCA vs. UDCA	1	−2.82 (−5.55 to −0.09)	-	-
Age of patients	≥55 years	7	−0.14 (−0.21 to −0.07)	20.1	0.276
​	<55 years	3	−0.18 (−0.23 to −0.13)	11.8	0.322
Sample size	≥1,000 participants	3	−0.16 (−0.31 to −0.01)	63.4	0.065
​	<1,000 participants	7	−0.16 (−0.20 to −0.11)	9.8	0.354
Duration of intervention	≥15 months	7	−0.19 (−0.23 to −0.14)	8.1	0.367
​	<15 months	3	−0.10 (−0.16 to −0.04)	0	0.778
Quality	Low	4	−0.17 (−0.25 to −0.08)	61.2	0.052
​	Moderate	3	−0.06 (−0.22 to 0.10)	0	0.593
​	High	3	−0.17 (−0.23 to −0.11)	0	0.386
ALT					
​	Overall	9	−9.09 (−14.69 to −3.48)	93.4	0.001
Type of intervention	Bezafibrate + UDCA vs. UDCA	6	−11.36 (−18.53 to −4.20)	95.9	0.001
​	Fenofibrate + UDCA vs. UDCA	3	−3.99 (−9.04 to 1.07)	0	0.936
Age of patients	≥55 years	6	−7.42 (−12.82 to −2.04)	90.3	0.001
​	<55 years	3	−12.54 (−25.44 to 0.36)	88.7	0.001
Sample size	≥1,000 participants	2	−5.84 (−14.08 to 2.41)	21.9	0.258
​	<1,000 participants	7	−9.53 (−15.95 to −3.12)	95	0.001
Duration of intervention	≥15 months	5	−9.65 (−17.32 to −1.97)	96.3	0.001
​	<15 months	4	−9.24 (−14.87 to −3.60)	36.8	0.191
Quality	Low	2	−15.32 (−25.46 to −5.18)	95.3	0.001
​	Moderate	4	−2.16 (−3.40 to −0.93)	0	0.728
​	High	3	−9.86 (−17.72 to −1.99)	65.4	0.055
AST					
​	Overall	5	−0.27 (−1.59 to 1.05)	0	0.423
Type of intervention	Bezafibrate + UDCA vs. UDCA	3	−0.15 (−1.53 to 1.23)	0	0.402
​	Fenofibrate + UDCA vs. UDCA	2	−1.66 (−7.64 to 4.33)	42.2	0.189
Age of patients	≥55 years	3	−0.32 (−3.96 to 3.32)	43.9	0.168
​	<55 years	2	0.47 (−4.82–5.75)	0	0.629
Sample size	≥1,000 participants	2	0.47 (−4.82–5.75)	0	0.629
​	<1,000 participants	3	−0.32 (−3.96 to 3.32)	43.9	0.168
Duration of intervention	≥15 months	3	−0.08 (−1.43 to 1.26)	0	0.571
​	<15 months	1	−4.89 (−11.65 to 1.87)	0	-
Quality	Low	1	4.53 (−2.54–11.60)	0	-
​	Moderate	2	−1.36 (−5.13 to 2.41)	40.8	0.194
​	High	2	0.47 (−4.82–5.75)	0	0.629
GGT					
​	Overall	13	−31.40 (−39.73 to −23.08)	92.9	0.001
Type of intervention	Bezafibrate + UDCA vs. UDCA	8	−26.37 (−34.57 to −18.18)	95	0.001
​	Fenofibrate + UDCA vs. UDCA	4	−72.17 (−100.70 to −43.64)	0	0.614
​	Fibrates (fenofibrate + bezafibrate) + UDCA vs. UDCA	1	−130.73 (−255.88 to −5.58)	-	-
Age of patients	≥55 years	9	−51.77 (−74.22 to −29.32)	93.8	0.001
​	<55 years	4	−44.20 (−93.07 to 4.68)	92.4	0.001
Sample size	≥1,000 participants	4	−38.40 (−81.15 to 4.56)	74.3	0.009
​	<1,000 participants	9	−54.14 (−76.79 to −31.49)	94.9	0.001
Duration of intervention	≥15 months	8	−21.12 (−29.46 to −12.79)	93	0.001
​	<15 months	5	−56.88 (−86.85 to −26.91)	81.7	0.001
Quality	Low	4	−71.51 (−108.31 to −34.72)	76.8	0.005
​	Moderate	5	−5.47 (−10.13 to −0.81)	84.2	0.001
​	High	4	−64.49 (−86.97 to −42.01)	33.6	0.211

RR (Relative Risk): Used for dichotomous outcomes (e.g., mortality, adverse events, pruritus rate).

MD (Mean Difference): Used for continuous outcomes (e.g., ALP, IgM, TG, bilirubin, ALT, AST, GGT), expressed in original clinical units. A negative MD, indicates a reduction in the outcome with fibrates + UDCA, compared to UDCA, alone.

I^2^, represents heterogeneity across studies.

P (Heterogeneity): Statistical significance of heterogeneity.

NR, not reported.

Standardized mean difference (SMD) values for ALP, extracted from meta-analyses that pooled heterogeneous measurement scales, are presented in Supplementary Table S3 for methodological transparency. These values should not be directly compared with MD estimates due to differences in scaling and interpretation.

##### ALT

All interventions significantly reduced ALT levels:

- UDCA: SMD: −35.67, 95% CI: −43.71 to −27.63
- Fibrates + UDCA: SMD: −9.09, 95% CI: −14.69 to −3.48
- Fibrate monotherapy: SMD: −12.80, 95% CI: −23.58 to −2.02

Substantial heterogeneity was evident for combination therapy (I^2^ = 93.4%, P = 0.001). Subgroup analysis revealed that ALT reduction was most pronounced in patients ≥55 years, primarily with bezafibrate + UDCA, whereas fenofibrate + UDCA did not show a comparable effect.

##### GGT

Combination therapy significantly reduced GGT levels (SMD: −31.40, 95% CI: −39.73 to −23.08), while other treatments had no significant effect. Subgroup analyses showed that GGT reduction with fibrates plus UDCA was especially notable in patients ≥55 years, with benefits observed for both bezafibrate and fenofibrate. UDCA monotherapy also reduced GGT in high-quality studies when treatment lasted ≥24 months.

##### AST

Only UDCA monotherapy significantly decreased AST levels (SMD: −35.65, 95% CI: −42.38 to −28.92) with minimal heterogeneity.

##### Bilirubin

All interventions significantly reduced bilirubin:

- UDCA: SMD: −9.31, 95% CI: −13.09 to −5.53
- Fibrate + UDCA: SMD: −0.16, 95% CI: −0.20 to −0.11
- Fibrate monotherapy: SMD: −0.19, 95% CI: −0.38 to −0.001

Subgroup analyses consistently demonstrated bilirubin-lowering efficacy of fibrates combined with UDCA across all examined subgroups (Table 3). Specifically:

- By fibrate type: Bezafibrate + UDCA (SMD: 0.20, 95% CI: 0.24 to −0.15; I^2^ = 0%, P = 0.960); Fenofibrate + UDCA (SMD: 0.10, 95% CI: 0.16 to −0.05; I^2^ = 0%, P = 0.919)
- By patient age: Patients ≥55 years (SMD: 0.14, 95% CI: 0.21 to −0.07; I^2^ = 20.1%, P = 0.276); Patients <55 years (SMD: 0.18, 95% CI: 0.23 to −0.13; I^2^ = 11.8%, P = 0.322)
- By treatment duration: ≥15 months (SMD: 0.19, 95% CI: 0.23 to −0.14; I^2^ = 8.1%, P = 0.367); <15 months (SMD: 0.10, 95% CI: 0.16 to −0.04; I^2^ = 0%, P = 0.778)
- By study quality: High quality (SMD: 0.17, 95% CI: 0.23 to −0.11; I^2^ = 0%, P = 0.386); Moderate quality (SMD: 0.06, 95% CI: 0.22 to 0.10; I^2^ = 0%, P = 0.593); Low quality (SMD: 0.17, 95% CI: 0.25 to −0.08; I^2^ = 61.2%, P = 0.052)

These findings confirm that the bilirubin-lowering effect of fibrate-UDCA combination therapy is robust across different patient populations, treatment durations, and fibrate types, with the effect being slightly more pronounced with bezafibrate and with longer treatment duration (≥15 months).

##### Albumin

Fibrate monotherapy increased serum albumin (SMD: 0.70, 95% CI: 0.61–0.79), while other interventions did not significantly alter albumin levels.

##### Immunoglobulin M (IgM)

All interventions significantly reduced IgM:

- UDCA: SMD: −1.32, 95% CI: −1.70 to −0.94
- Fibrate + UDCA: SMD: −36.84, 95% CI: −44.25 to −29.43
- Fibrate monotherapy: SMD: −79.37, 95% CI: −152.53 to −6.21

High heterogeneity was observed for combination therapy. Subgroup analysis showed efficacy in patients ≥55 years, for both bezafibrate and fenofibrate combined with UDCA.

##### Triglycerides and cholesterol

**FIGURE 4:**
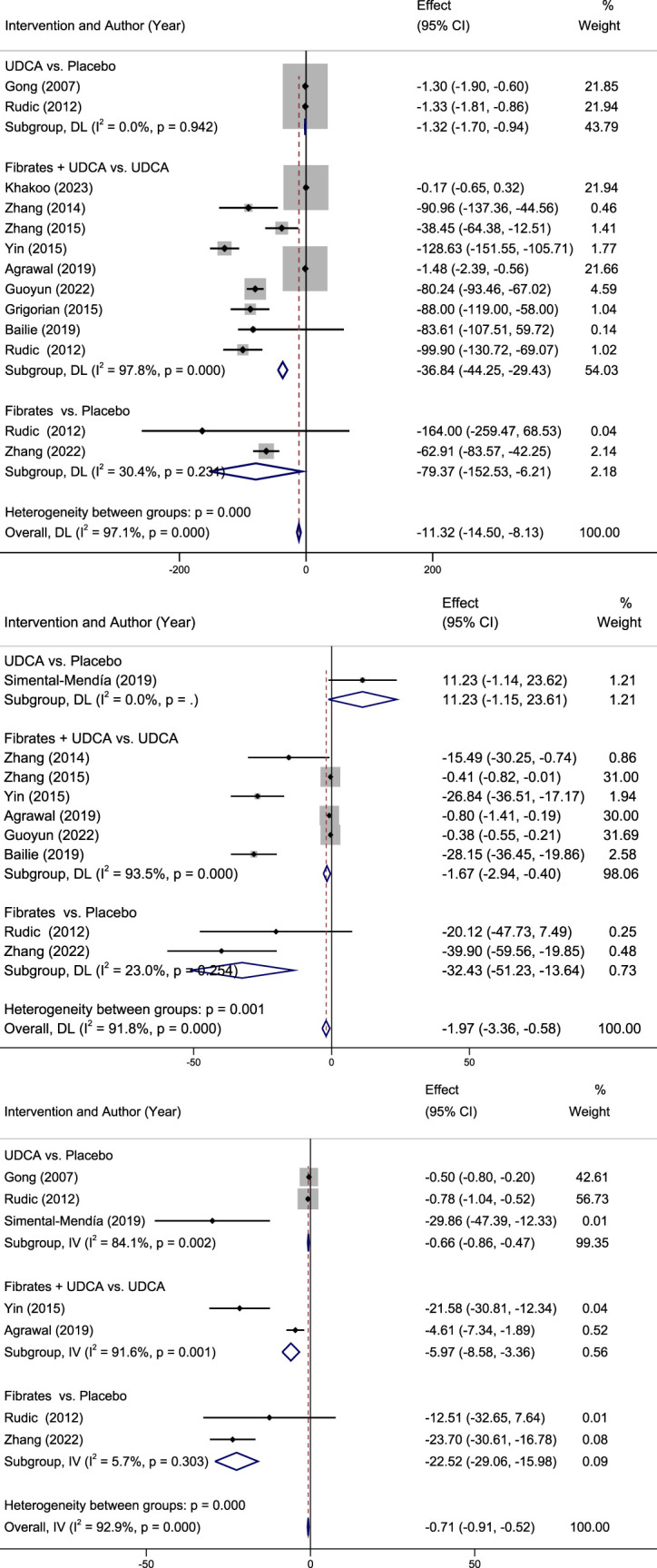
Meta-analysis of the effects of UDCA monotherapy, fibrate monotherapy, and combination therapy on additional biochemical markers in patients with PBC: **(A)** immunoglobulin M (IgM), **(B)** triglycerides, **(C)** total cholesterol.

Fibrates, alone or combined with UDCA, significantly reduced TG, whereas UDCA monotherapy showed no effect. All interventions significantly lowered TC (Figure 4).

##### Publication bias, sensitivity, and meta-regression

Publication bias was evident for ALP, GGT, and IgM in the fibrate versus placebo comparison (Egger’s test: ALP P = 0.001, GGT P = 0.001, IgM P = 0.003). Trim-and-fill analyses indicated that pooled effect sizes remained stable, confirming the robustness of the results. Sensitivity analyses demonstrated that no individual study influenced the pooled effects of combination therapy. Meta-regression showed that pooled effect sizes were not significantly influenced by age, sample size, study quality, or follow-up duration.

## Discussion

This umbrella meta-analysis synthesized evidence across three therapeutic strategies in PBC: UDCA vs. placebo, fibrates vs. placebo, and combination therapy vs. UDCA. UDCA demonstrated modest biochemical improvement, fibrates showed substantial reductions in cholestatic markers, and combination therapy yielded the greatest biochemical response. Clinically, these findings support the role of fibrates as effective second-line agents and highlight the enhanced efficacy of combination therapy in patients with inadequate UDCA response. When biochemical outcomes were analyzed using Mean Differences in original clinical units, UDCA demonstrated modest ALP reduction (−32.1 U/L), fibrates showed moderate reductions (−48.7 U/L), and combination therapy yielded the greatest additional benefit (−85.4 U/L beyond UDCA alone). These absolute reductions are clinically meaningful: the combination therapy effect exceeds the ≥40 U/L threshold associated with improved long-term outcomes per Paris II criteria (Lammers et al., 2014). Similarly, GGT reduction with UDCA was only evident in high-quality studies with follow-up ≥24 months. For combination therapy, bilirubin reduction was more pronounced with treatment duration ≥15 months (SMD: 0.19, 95% CI: 0.23 to −0.14) compared to shorter durations (SMD: 0.10, 95% CI: 0.16 to −0.04). Regarding baseline disease severity, patients with higher baseline ALP and bilirubin levels (indicative of more advanced disease) showed greater absolute reductions with combination therapy, although relative improvements were consistent across severity strata. These findings underscore the importance of patient selection in therapeutic decision-making, suggesting that prolonged UDCA therapy is necessary to observe biochemical benefits, while combination therapy may be particularly advantageous for patients with inadequate UDCA response or more advanced biochemical abnormalities.

The absence of a statistically significant mortality benefit for UDCA or combination therapy in this umbrella meta-analysis requires careful clinical interpretation. PBC is a slowly progressive disease with a natural history spanning decades, and the long-term survival benefit of UDCA has been well-documented in extended follow-up studies and large observational cohorts (Trivella et al., 2023). The randomized controlled trials included in the constituent meta-analyses had relatively short follow-up periods (mean duration 8–39 months), which are insufficient to capture differences in hard clinical endpoints such as mortality or liver transplantation. Furthermore, the event rates for mortality were low across all trials, limiting statistical power to detect differences between treatment groups. Therefore, the absence of a statistically significant mortality reduction should not be interpreted as absence of disease-modifying efficacy. Rather, it reflects the inherent limitations of short-term RCTs in assessing long-term outcomes in slowly progressive diseases. Biochemical markers, particularly bilirubin and ALP, have emerged as validated surrogate endpoints that correlate strongly with long-term outcomes (Lammers et al., 2014; Poupon, 2010), and the significant improvements observed with combination therapy on these markers suggest potential for improved clinical outcomes with prolonged treatment.

Our findings align with previous meta-analyses reporting significant ALP and GGT reduction with fibrate therapy, particularly when combined with UDCA (Feng et al., 2019; Grigorian et al., 2015; Roglans et al., 2007). However, unlike earlier reviews that focused on single comparisons, this umbrella synthesis highlights the differential magnitude of effect across three therapeutic strategies and underscores the variability in reported response definitions across studies.

From a biochemical perspective, fibrates combined with UDCA significantly lowered pruritus scores, ALP, GGT, and TG levels, suggesting their role as optimal adjuncts for UDCA non-responders (Feng et al., 2019; Grigorian et al., 2015). Importantly, all therapies demonstrated excellent safety profiles, supporting their long-term use in PBC management. In the present study, the beneficial effects of UDCA monotherapy on outcomes such as ALP and GGT were observed primarily with prolonged treatment. Furthermore, the combination of fibrates and UDCA demonstrated enhanced effects particularly in older patients, with bezafibrate—but not fenofibrate—showing significant ALT reductions in patients aged ≥55 years. Both bezafibrate and fenofibrate combined with UDCA significantly reduced GGT and IgM levels in older patients. These observations suggest that long-term UDCA monotherapy is effective for certain biomarkers, whereas selective fibrate combination therapy provides enhanced biochemical benefits, especially in older patients, depending on the fibrate type used. Collectively, these findings reinforce UDCA as the cornerstone of PBC therapy, with fibrates serving as effective adjuncts for patients with suboptimal biochemical response or persistent pruritus, emphasizing the need for personalized, biomarker-driven treatment approaches to optimize outcomes.

Assessing the impact of UDCA-fibrate combination therapy on critical clinical endpoints, such as histological improvements and liver transplantation rates, remains essential. However, the slow progression of PBC, limited patient availability, and relatively short study durations pose challenges for evaluating these outcomes (Agrawal et al., 2019). Most studies included in this analysis did not provide sufficient data on these endpoints. Nonetheless, biochemical markers, particularly bilirubin and ALP, have emerged as reliable predictors of long-term outcomes, including liver transplantation and survival (Grigorian et al., 2015). Serum bilirubin and ALP levels negatively correlate with survival (Lammers et al., 2014; Poupon, 2010), indicating that reductions observed with combination therapy may potentially translate into improved major clinical outcomes.

Mechanistically, fibrate-UDCA combination therapy operates through complementary pathways targeting cholestasis, inflammation, and lipid metabolism. Fibrates primarily activate PPAR-α, β/δ, and γ receptors, which upregulate MDR3, a key transporter facilitating phospholipid secretion (Honda et al., 2013; Staels et al., 1998; Ghonem et al., 2014; Kok et al., 2003; Ros et al., 2003). Activation of PPAR-α enhances lipoprotein lipase activity and fatty acid oxidation, leading to reductions in serum TC and TG (Staels et al., 1998). Fibrates also mitigate bile acid toxicity and reduce pro-inflammatory cytokine production, thereby slowing fibrosis progression (Agrawal et al., 2019; Roglans et al., 2007). In contrast, UDCA acts by balancing hydrophilic and hydrophobic bile acids and promoting bile acid, cholesterol, and phospholipid secretion (Poupon, 2012; Guoyun et al., 2022). Since the mechanisms of UDCA and fibrates are distinct yet complementary, their combination may produce synergistic benefits, particularly for UDCA-refractory patients, by enhancing mitochondrial function and reducing oxidative stress (Guoyun et al., 2022).

To the best of our knowledge, this is the first umbrella meta-analysis evaluating fibrates and UDCA in PBC. Strengths of this study include a comprehensive assessment of multiple biochemical endpoints, providing a holistic overview of treatment efficacy, and the identification of age-dependent responses (e.g., greater fibrate-UDCA efficacy in patients aged ≥55 years), supporting personalized therapy approaches. Observed tolerability across all therapies is consistent with previous meta-analyses (Yin et al., 2015; Zhu et al., 2015), reinforcing their suitability for long-term management.

As an umbrella meta-analysis, this study synthesized evidence at the level of published meta-analyses rather than re-analyzing primary RCT data. This approach offers the advantage of providing a comprehensive overview of the existing evidence base while leveraging the rigorous methodology of previously published systematic reviews. However, it also introduces potential limitations, including dependence on the quality of included meta-analyses, inability to standardize outcome definitions across reviews, and potential duplication of primary studies. We addressed these limitations through rigorous quality assessment using AMSTAR-2, calculation of study overlap using CCA, and sensitivity analyses to explore heterogeneity. Nonetheless, some limitations should be acknowledged. Significant heterogeneity across included studies may limit the generalizability of findings. Subgroup analyses indicated that fibrate type, treatment duration, patient age, study quality, and sample size contributed to this variability. Small numbers of studies in certain subgroup analyses may skew efficacy estimates or produce false-negative results, warranting cautious interpretation. Limitation regarding statistical artifacts in primary meta-analyses: This umbrella review identified one low-quality meta-analysis (Grigorian et al., 2015) that reported statistically implausible SMD values for ALP reduction (−226.07), likely due to inappropriate use of standard error rather than standard deviation in the SMD calculation. This value was excluded from our pooled estimates, and only methodologically sound meta-analyses reporting mean differences were retained. Another limitation relates to subgroup analyses, which could not be uniformly conducted across all outcomes. This was primarily due to inconsistent reporting of subgroup-level estimates in the original meta-analyses, particularly for clinically important endpoints such as mortality. Consequently, subgroup findings should be interpreted within the context of data availability rather than selective analytical choice. This incident highlights a broader challenge in umbrella reviews: the propagation of statistical errors from primary meta-analyses. To mitigate this risk, we implemented a verification protocol whereby any effect size exceeding |SMD| > 5 was traced to its original source and validated. Future umbrella reviews should consider incorporating such verification steps as standard practice. Evidence of publication bias for some outcomes suggests that smaller studies may have been missed. This umbrella review was not prospectively registered in PROSPERO or other systematic review registries. The lack of prospective registration is acknowledged as a limitation of this study. Another important limitation of the current evidence base, reflected in the included meta-analyses, is the absence of data on validated prognostic models such as the GLOBE and UK-PBC scores. These scores integrate multiple biochemical variables (including ALP, bilirubin, and albumin) to predict transplant-free survival and have become standard tools for risk stratification in contemporary PBC management (Trivella et al., 2023). None of the included meta-analyses reported treatment effects on GLOBE or UK-PBC scores, as the primary studies did not collect or report these composite outcomes. This gap limits our ability to assess whether the biochemical improvements observed with combination therapy translate into improved predicted long-term outcomes according to validated risk models. Future RCTs should incorporate these prognostic scores as secondary endpoints to better characterize the clinical relevance of biochemical responses and to enable more nuanced risk-benefit assessments of combination therapy. Finally, reliance on surrogate biochemical markers rather than histological endpoints may limit the direct clinical applicability of results, reflecting broader challenges in PBC research, including the need for standardized prognostic tools and validated long-term endpoints. Future research should consider individual patient data meta-analysis to further refine effect estimates and identify patient-level predictors of treatment response.

## Conclusion

In conclusion, while fibrate monotherapy, UDCA monotherapy, and their combination therapy improved biochemical markers in PBC patients, no mortality benefit was observed. The findings support UDCA-fibrate combination therapy as a more effective approach than UDCA alone for improving biochemical and symptomatic outcomes. Further prospective randomized trials are warranted to assess long-term clinical benefits and safety, which are essential to optimize treatment strategies and improve prognosis in this patient population.

## Data Availability

The original contributions presented in the study are included in the article/Supplementary Material, further inquiries can be directed to the corresponding author.
