# Supplementary material for: Comparative efficacy and safety of ursodeoxycholic acid, fibrates, and combination therapy in primary biliary cholangitis: an umbrella meta-analysis of meta-analyses
Source: Front Pharmacol. 2026 May 25;17:1797227. doi: 10.3389/fphar.2026.1797227 (PMC13243085; doi:10.3389/fphar.2026.1797227)
Supplement: Supplementary file 1 [file Supplementaryfile1.docx]

| Section | Item | PRISMA Item Description | Location in Manuscript |
| --- | --- | --- | --- |
| TITLE | 1 | Identify the report as a systematic review | Title page |
| ABSTRACT | 2 | Structured summary including background, methods, results, conclusions | Abstract |
| INTRODUCTION | 3 | Rationale for the review in context of existing knowledge | Introduction, paragraphs 3–5 |
|  | 4 | Explicit statement of objectives or questions (PICO) | End of Introduction |
| METHODS | 5 | Eligibility criteria (PICOS) | Methods – Selection Criteria |
|  | 6 | Information sources (databases, dates) | Methods – Search Strategy |
|  | 7 | Full search strategy | Methods – Search Strategy |
|  | 8 | Selection process (number of reviewers, independence) | Methods – Study Selection |
|  | 9 | Data collection process | Methods – Data Extraction |
|  | 10 | Data items sought | Methods – Data Extraction |
|  | 11 | Risk of bias assessment | Methods – Quality Assessment (AMSTAR-2) |
|  | 12 | Effect measures (SMD, RR, CI) | Methods – Statistical Analysis |
|  | 13 | Synthesis methods (random effects, heterogeneity, subgroups, meta-regression) | Methods – Statistical Analysis |
|  | 14 | Reporting bias assessment (Egger, funnel, trim-fill) | Methods – Statistical Analysis |
|  | 15 | Certainty of evidence (GRADE) | Methods – added paragraph after PRISMA statement |
| RESULTS | 16 | Study selection with numbers (PRISMA flow) | Results – Study Selection + Figure 1 |
|  | 17 | Characteristics of included studies | Table 1 |
|  | 18 | Risk of bias of included studies | Table 1 (AMSTAR-2 ratings) |
|  | 19 | Results of individual studies | Forest plots (Figures 2–4) |
|  | 20 | Results of syntheses for each comparison | Results – Meta-analysis sections |
|  | 21 | Reporting bias results | Results – Publication Bias |
|  | 22 | Certainty of evidence results | Supplementary Table (GRADE) |
| DISCUSSION | 23 | Summary of evidence | Discussion – first 3 paragraphs (restructured) |
|  | 24 | Limitations of evidence | Discussion – limitations paragraph |
|  | 25 | Interpretation and clinical implications | Discussion – clinical implications |
| OTHER INFORMATION | 26 | Registration and protocol | Methods – PRISMA paragraph (PROSPERO added) |
|  | 27 | Support/funding | Funding statement |
|  | 28 | Competing interests | Conflict of interest statement |
|  | 29 | Availability of data/materials | Data availability statement |
