# Supplementary material for: Comparative efficacy and safety of ursodeoxycholic acid, fibrates, and combination therapy in primary biliary cholangitis: an umbrella meta-analysis of meta-analyses
Source: Front Pharmacol. 2026 May 25;17:1797227. doi: 10.3389/fphar.2026.1797227 (PMC13243085; doi:10.3389/fphar.2026.1797227)
Supplement: Supplementary file 2 [file Table1.docx]

| **Table S1. AMSTAR-2 Quality Assessment of Included Meta-Analyses** | | | | | | | | | | | | | | | | | | | | | | | | |
| --- | --- | --- | --- | --- | --- | --- | --- | --- | --- | --- | --- | --- | --- | --- | --- | --- | --- | --- | --- | --- | --- | --- | --- | --- |
| **Study** | **Year** | **Country** | **No. of Studies** | **Sample Size** | **Intervention(s)/Control** | **Bias Tool (High Quality/Total)** | **D1** | **D2** | **D3** | **D4** | **D5** | **D6** | **D7** | **D8** | **D9** | **D10** | **D11** | **D12** | **D13** | **D14** | **D15** | **D16** | **Overall AMSTAR-2 Rating** | **Outcomes Assessed** |
| Khakoo | 2023 | USA | 7 | 279 | Bezafibrate + UDCA vs. UDCA | Cochrane, 0/8 | Y | Y | Y | Y | Y | Y | Y | Y | Y | Y | Y | Y | Y | Y | Y | Y | High | ALP, GGT, IgM, TB, pruritus rate |
| Zhang | 2015 | China | 7 | 171 | Bezafibrate + UDCA vs. UDCA | Cochrane, 3/7 | Y | Y | PY | Y | Y | Y | PY | Y | Y | Y | Y | Y | PY | Y | Y | PY | Moderate | ALT, ALP, GGT, TG, IgM, AE, pruritus rate, mortality |
| Zhang | 2015 | China | 6 | 84 | Fenofibrate + UDCA vs. UDCA | Cochrane, 1/6 | Y | Y | PY | Y | Y | Y | PY | Y | Y | Y | Y | Y | PY | Y | Y | PY | Moderate | ALT, ALP, GGT, TG, IgM, TB, AE, pruritus rate |
| Yin | 2015 | China | 9 | 269 | Bezafibrate + UDCA vs. UDCA | Cochrane, 2/9 | Y | PY | N | Y | Y | Y | N | Y | Y | Y | Y | Y | N | Y | Y | N | Low | ALT, ALP, GGT, TG, IgM, TC, TB, AST, albumin, AE, pruritus rate, mortality |
| Agrawal | 2019 | USA | 10 | 369 | Bezafibrate + UDCA vs. UDCA | Cochrane, 6/10 | Y | Y | PY | Y | Y | Y | Y | Y | Y | Y | Y | Y | PY | Y | Y | Y | Moderate | ALT, ALP, GGT, TG, IgM, TC, TB, AST, albumin, AE, pruritus rate, fatigue, mortality |
| Guoyun | 2022 | China | 9 | 389 | Fenofibrate + UDCA vs. UDCA | Cochrane, 2/9 | Y | Y | PY | Y | Y | Y | PY | Y | Y | Y | Y | Y | Y | Y | Y | PY | Moderate | ALT, ALP, GGT, TG, IgM, TB, AST, AE, pruritus rate |
| Grigorian | 2015 | USA | 6 | 102 | Fenofibrate + UDCA vs. UDCA | NR | Y | PY | N | Y | Y | Y | N | Y | Y | Y | Y | Y | N | Y | Y | N | Low | ALP, GGT, IgM, TB |
| Zhu | 2015 | NR | 16 | 4182 | UDCA vs. Placebo; Bezafibrate + UDCA vs. UDCA | Cochrane, 12/16 | Y | Y | PY | Y | Y | Y | Y | Y | Y | Y | Y | Y | PY | Y | Y | Y | Moderate | AE, mortality |
| Lin | 2024 | China | 23 | 1734 | Bezafibrate + UDCA vs. UDCA; Fenofibrate + UDCA vs. UDCA | Cochrane, NR | Y | Y | PY | Y | Y | Y | Y | Y | Y | Y | Y | Y | PY | Y | Y | Y | Moderate | ALP |
| Zhang | 2022 | China | 20 | 4783 | Fibrates + UDCA vs. UDCA; Bezafibrate vs. Placebo | NR | Y | PY | N | Y | Y | Y | N | Y | Y | Y | Y | Y | N | Y | Y | N | Low | GGT, TB, pruritus rate, mortality, ALT, AST, ALP, TG, IgM, TC, albumin |
| Bailie | 2019 | China | 11 | 465 | Bezafibrate + UDCA vs. UDCA | NR | Y | PY | N | Y | Y | Y | N | Y | Y | Y | Y | Y | N | Y | Y | N | Low | ALT, ALP, GGT, TG, IgM, TB, AE, pruritus score, mortality |
| Saffioti | 2017 | UK | 24 | 4274 | UDCA vs. Placebo; Bezafibrate + UDCA vs. UDCA | Cochrane, 2/24 | Y | Y | Y | Y | Y | Y | Y | Y | Y | Y | Y | Y | Y | Y | Y | Y | High | Mortality |
| Saeedian | 2025 | Iran | 8 | 7795 | Bezafibrate + UDCA vs. UDCA; Fenofibrate + UDCA vs. UDCA | Cochrane, 2/8 | Y | Y | Y | Y | Y | Y | Y | Y | Y | Y | Y | Y | Y | Y | Y | Y | High | ALT, ALP, GGT, TB, AST, AE, pruritus rate |
| Xu | 2022 | China | 10 | 1191 | UDCA vs. Placebo; Bezafibrate + UDCA vs. UDCA | Cochrane, 8/10 | Y | Y | PY | Y | Y | Y | Y | Y | Y | Y | Y | Y | PY | Y | Y | Y | Moderate | ALP, GGT, AE, pruritus rate, pruritus score |
| Rudic | 2012 | Serbia | 6 | 151 | Bezafibrate + UDCA vs. UDCA; Bezafibrate vs. Placebo | Cochrane, 0/6 | Y | Y | Y | Y | Y | Y | Y | Y | Y | Y | Y | Y | Y | Y | Y | Y | High | ALT, ALP, GGT, IgM, mortality, TG, TC, TB, AE, pruritus rate |
| Lee | 2019 | USA | 6 | 952 | UDCA vs. Placebo | Cochrane, 5/6 | Y | Y | PY | Y | Y | Y | Y | Y | Y | Y | Y | Y | PY | Y | Y | Y | Moderate | Fatigue, AE |
| Gong | 2007 | Denmark | 16 | 1447 | UDCA vs. Placebo | Cochrane, 9/16 | Y | Y | PY | Y | Y | Y | Y | Y | Y | Y | Y | Y | PY | Y | Y | Y | Moderate | ALT, ALP, GGT, IgM, TC, TB, AST, AE, pruritus rate, pruritus score, fatigue, mortality |
| Rudic | 2012 | Serbia | 16 | 1447 | UDCA vs. Placebo | Cochrane, 12/16 | Y | Y | Y | Y | Y | Y | Y | Y | Y | Y | Y | Y | Y | Y | Y | Y | High | ALT, ALP, GGT, IgM, TC, TB, AST, albumin, AE, pruritus rate, pruritus score, fatigue, mortality |
| Goulis | 1999 | UK | 11 | 1272 | UDCA vs. Placebo | NR | Y | PY | N | Y | Y | Y | N | Y | Y | Y | Y | Y | N | Y | Y | N | Low | AE, mortality |
| Shi | 2006 | China | 7 | 1038 | UDCA vs. Placebo | Jadad, 6/7 | Y | Y | PY | Y | Y | Y | Y | Y | Y | Y | Y | Y | PY | Y | Y | Y | Moderate | AE, mortality |
| Simental-Mendía | 2019 | Iran | 15 | 1370 | UDCA vs. Placebo | Cochrane, 6/15 | Y | Y | PY | Y | Y | Y | Y | Y | Y | Y | Y | Y | PY | Y | Y | Y | Moderate | TG, TC |
| Medina-Morales | 2023 | USA | 11 | 1002 | UDCA vs. Placebo; Bezafibrate vs. Placebo | Cochrane, 2/11 | Y | Y | Y | Y | Y | Y | Y | Y | Y | Y | Y | Y | Y | Y | Y | Y | High | AE, pruritus score |
| Shen | 2021 | China | 7 | 382 | Bezafibrate vs. Placebo; Fenofibrate vs. Placebo | Cochrane, 2/7 | Y | PY | N | Y | Y | Y | N | Y | Y | Y | Y | Y | N | Y | Y | N | Low | AE, pruritus rate, pruritus score |
| AMSTAR-2 Domains: D1: Protocol registered prior to study commencement; D2: Adequate literature search (e.g., multiple databases, no language restrictions); D3: Justification for excluding studies; D4: Risk of bias assessment for included studies; D5: Appropriate meta-analysis methods; D6: Consideration of risk of bias in results interpretation; D7: Explanation of heterogeneity sources; D8: Adequate reporting of study characteristics; D9: Risk of bias considered in discussion; D10: Funding sources reported for included studies; D11: Appropriate statistical methods for meta-analysis; D12: Impact of risk of bias on meta-analysis results; D13: Publication bias assessment (e.g., Egger’s test, funnel plots); D14: Conflict of interest statement provided; D15: Investigation of heterogeneity (e.g., subgroup analysis, meta-regression); D16: Reporting of funding sources for the review; Y: Yes; PY: Partial Yes; N: No. Overall AMSTAR-2 Rating: High: No or one non-critical weakness; Moderate: More than one non-critical weakness; Low: One critical flaw (e.g., D2, D4, D7, D9, D11, D13, D15) with or without non-critical weaknesses. Abbreviations: ALP: Alkaline phosphatase; ALT: Alanine aminotransferase; AST: Aspartate aminotransferase; GGT: Gamma-glutamyltransferase; TB: Total bilirubin; TG: Triglycerides; IgM: Immunoglobulin M; AE: Adverse events; UDCA: Ursodeoxycholic acid; NR: Not reported. Bias Tool: Indicates the tool used for risk of bias assessment (Cochrane or Jadad) and the proportion of high-quality studies as reported in Table 1. | | | | | | | | | | | | | | | | | | | | | | | | |
