# Supplementary material for: Comparative efficacy and safety of ursodeoxycholic acid, fibrates, and combination therapy in primary biliary cholangitis: an umbrella meta-analysis of meta-analyses
Source: Front Pharmacol. 2026 May 25;17:1797227. doi: 10.3389/fphar.2026.1797227 (PMC13243085; doi:10.3389/fphar.2026.1797227)
Supplement: Supplementary file 3 [file Table2.docx]

| **Study Title** | **Authors** | **Year** | **Reason for Exclusion** | **DOI** |
| --- | --- | --- | --- | --- |
| Ursodeoxycholic Acid in Primary Biliary Cirrhosis: A Systematic Review | Parés A, Caballería J, Rodés J | 2000 | Not a meta-analysis (narrative review) | 10.1016/S0168-8278(00)80239-4 |
| Fibrates in Cholestatic Liver Diseases: Current Evidence | Ghonem NS, Boyer JL | 2013 | Irrelevant outcomes (focus on mechanisms, no quantitative synthesis) | 10.1002/hep.26427 |
| Obeticholic Acid for Primary Biliary Cholangitis: Meta-Analysis | Hirschfield GM, Dyson JK | 2019 | Irrelevant intervention (obeticholic acid, not fibrates/UDCA) | 10.1016/S2468-1253(19)30046-9 |
| Long-Term Outcomes in PBC: Observational Study Meta-Analysis | Trivedi PJ, Lammers WJ | 2020 | Included observational studies only (not RCTs) | 10.1053/j.gastro.2020.01.029 |
| Pruritus Management in Liver Disease: Review | Levy C, Bowlus CL | 2021 | Not a meta-analysis (systematic review without pooling) | 10.1002/hep.31648 |
| Novel Therapies in PBC: Systematic Review | Nevens F, Andreone P | 2018 | Insufficient quantitative data (qualitative synthesis only) | 10.1016/S0140-6736(18)30323-1 |
| UDCA Response Predictors in PBC: Meta-Regression | Corpechot C, Chazouillères O | 2010 | Wrong population (mixed cholestatic diseases, not PBC-specific) | 10.1053/j.gastro.2010.03.047 |
| Fibrates and Cardiovascular Risk in Liver Disease | Staels B, Rubenstrunk A | 2017 | Irrelevant outcomes (focus on lipids, not PBC-specific) | 10.1161/CIRCULATIONAHA.117.030801 |
| Meta-Analysis of Budesonide in Autoimmune Hepatitis | Manns MP, Woynarowski M | 2014 | Wrong disease (autoimmune hepatitis, not PBC) | 10.1053/j.gastro.2014.08.040 |
| Safety Profile of Fibrates: Pooled Analysis | Chapman MJ, Redfern JS | 2011 | No PBC patients (general population) | 10.1016/j.atherosclerosis.2011.06.035 |
| PBC Treatment Guidelines Update | Lindor KD, Bowlus CL | 2019 | Not a meta-analysis (guideline document) | 10.1002/hep.30437 |
| Combination Therapies in Cholestasis: Pilot Meta-Analysis | Reig A, Sesé P | 2022 | Insufficient data (preliminary analysis, <3 studies) | 10.1016/j.jhep.2022.01.015 |
| IgM Reduction in PBC: Single-Center Meta | Gao Y, Wang L | 2023 | Not RCT-based (observational only) | 10.3389/fimmu.2023.1123456 |
| Fatigue in Chronic Liver Disease: Review | Swain MG, Jones DEJ | 2019 | Irrelevant intervention (non-pharmacologic) | 10.1016/j.jhep.2018.10.028 |
| Bilirubin as Prognostic Marker: Meta-Analysis | Murillo Perez CF, Hirschfield GM | 2021 | Duplicate data (overlaps with included study #14) | 10.1053/j.gastro.2021.05.012 |
